# Two Months of Active Video Game Training Improves Selected Lipid Profile Markers in Older Adults: A Preliminary Study

**DOI:** 10.3390/geriatrics11030052

**Published:** 2026-04-23

**Authors:** Agali Y. López-Miguel, Ángel E. Brizuela-Araujo, Omar A. López-López, Juan J. Calleja-Núñez, Roberto Espinoza-Gutiérrez, Elena C. Guzmán-Gutiérrez, Aracely Serrano-Medina, José Moncada-Jiménez, Jorge A. Aburto-Corona

**Affiliations:** 1 Faculty of Sports, Autonomous University of Baja California, Tijuana 22427, Mexicoespinoza.roberto@uabc.edu.mx (R.E.-G.); cecilia.guzman@uabc.edu.mx (E.C.G.-G.); 2 Research Group UABC-CA-341 Physical Performance and Health, Human Motor Sciences Biosciences Laboratory, Tijuana 22427, Mexico; 3 Interuniversity Network for Healthy Aging, Latin America and the Caribbean (RIES-LAC), Medellín, Colombia; 4 Faculty of Medicine and Psychology, Autonomous University of Baja California, Tijuana 22427, Mexico; serrano.aracely@uabc.edu.mx; 5 Human Movement Sciences Research Center (CIMOHU), University of Costa Rica, San Jose 2060, Costa Rica; jose.moncada@ucr.ac.cr

**Keywords:** exergames, serious games, blood glucose, cholesterol

## Abstract

**Background**: The purpose of this study was to compare the effects of two months of exergaming, conventional resistance exercise training, and no exercise on body composition and cardiometabolic risk factors in physically inactive older adults. **Methods**: For the preliminary study, twenty-four physically inactive adults aged 60–74 yrs. were allocated to an active video game training group (AVG n = 8), a conventional exercise group (CEG n = 7), or a non-exercising control group (CON n = 9). The AVG and CEG completed 24 supervised exercise training sessions over two months (three sessions per week) at self-selected, predominantly moderate-to-vigorous intensity, while the CON maintained usual daily activities. Body weight, skeletal muscle mass, body fat percentage, phase angle, and fasting blood biomarkers (glucose, total cholesterol, LDL, HDL, VLDL, and triglycerides) were assessed before and after the intervention. **Results**: No significant interactions were observed for body composition variables. Body weight decreased significantly following exercise training in both the AVG and CEG (*p* < 0.05). Significant interactions were found for total cholesterol (*p* = 0.001) and LDL cholesterol (*p* = 0.009). The AVG demonstrated significant reductions in fasting glucose, total cholesterol, and LDL cholesterol (*p* < 0.05), whereas the CEG showed a significant reduction only in total cholesterol. In contrast, the CON exhibited a significant increase in total cholesterol over the same period (*p* < 0.05). **Conclusions**: Two months of exergaming-based exercise training may lead to greater improvements in lipid-related cardiometabolic risk factors compared with conventional resistance exercise training in physically inactive older adults. These findings suggest that exergaming could be a promising exercise modality for supporting cardiometabolic health in aging populations.

## 1. Introduction

Population aging constitutes one of the major demographic and public health challenges worldwide. In recent decades, the proportion of individuals aged 60 years and older has increased steadily (from 12% in 2015 to 22% projected for 2050), and this trend is expected to continue accelerating in the coming years due to increased life expectancy and declining birth rates [1,2]. This demographic shift is associated with a rising prevalence of non-communicable chronic diseases, representing a significant burden on health systems and underscoring the need for effective and sustainable preventive strategies targeting this population [3].

The aging process is accompanied by multiple physiological changes that affect the metabolic and functional health of older adults. Among these changes, alterations in body composition are particularly noteworthy, characterized not only by a progressive decline in muscle mass, but also by an increase in fat mass, as well as deleterious modifications in the lipid profile, including elevations in total cholesterol, low-density lipoproteins, and triglycerides, along with a reduction in high-density lipoproteins [4]. These alterations contribute to an increased cardiovascular, metabolic, and functional risk, and are closely associated with the loss of independence and quality of life in advanced age. Physical inactivity is one of the main modifiable risk factors associated with the development and progression of chronic diseases in older adults. Several international reports indicate that a considerable proportion of older individuals (18% to 45% of the older population) do not meet the minimum physical activity recommendations. The lack of sufficient daily physical activity is closely associated with a high prevalence of dyslipidemia, obesity, type 2 diabetes, and hypertension [5,6,7]. In this context, the promotion of regular physical exercise is recognized as a fundamental non-pharmacological intervention for the prevention and management of these conditions in the aging population [7,8].

Among the different exercise training modalities, resistance training is particularly effective to maintaining or increasing muscle mass in older adults. Indeed, reductions in fat mass and improvements in various metabolic markers, including the lipid profile, have been reported in this age group [9]. However, despite the evidence supporting its benefits, adherence to traditional exercise programs may be limited by factors such as lack of motivation, limited access to appropriate training facilities, or perceived monotony, thereby reducing their long-term effectiveness [10].

Active video games (as known as exergames) have emerged as an innovative alternative to traditional exercise training, aiming to promote physical activity by combining significant physical movement with playful, interactive stimuli. This modality has shown potential to enhance motivation, enjoyment, and adherence to exercise, particularly among populations facing barriers to conventional physical activity [11]. In older adults, recent studies have reported benefits of active video games on functional, cognitive, and cardiovascular outcomes; however, evidence regarding their effects on the lipid profile and body composition remains limited, and few studies have directly compared this modality with structured resistance training programs and control groups [12,13,14].

Based on the previous context, it is relevant to evaluate and compare the effects of different training modalities on key cardiometabolic health indicators in the older adult population. Therefore, this study aimed to determine whether two months of active video game training improved body weight, body composition (muscle mass and body fat), phase angle, and glucose and lipid profile (total cholesterol, low-density lipoprotein [LDL], high-density lipoprotein [HDL], very-low-density lipoprotein [VLDL], and triglycerides) compared with conventional resistance exercise and a control group of physically inactive older adults. It is hypothesized that training with active video games may improve cardiometabolic health indicators in inactive older adults, compared with conventional resistance training and no intervention.

## 2. Materials and Methods

Participants were recruited from a physical activity program for older adults entitled “Successful Aging: More Strength, More Life” offered by the Faculty of Sports Tijuana at the Autonomous University of Baja California, as well as from a senior day care center called “Day Center for Grandparents United by Love”. All participants invited to the project were newly enrolled in the program. The inclusion criteria were: (1) being between 60 and 74 yr. old; (2) being physically inactive (<600 METs-min/week); (3) having no musculoskeletal disorders; (4) having no prior experience with active video game exercise; and (5) having no cardiovascular, pulmonary, renal disease, diabetes, or any medical contraindication to performing physical exercise. This study was performed in accordance with the ethical guidelines of the Declaration of Helsinki and were reviewed and approved by the Evaluation Committee for Research and Postgraduate Studies from Autonomous University of Baja California, Mexico (code P02-2025-1; 28 February 2025).

**Figure 1:**
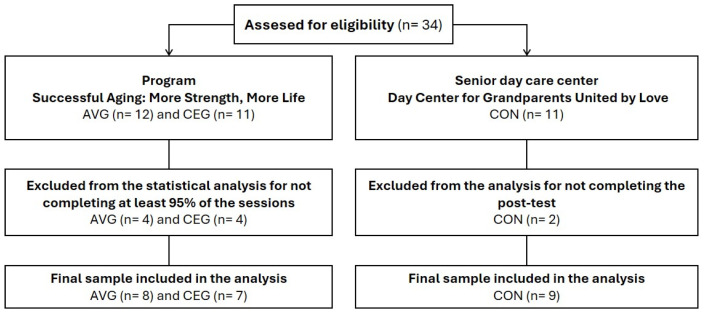
Flowchart of the recruitment process.

Participants in the AVGs and CEGs were recruited from the Faculty of Sport program, having recently enrolled and meeting criteria for physical inactivity. The control group was recruited from a local senior day care center, where no structured physical activity was offered. This approach ensured ethical compliance, avoiding the exclusion of older adults from potential health benefits of physical activity. Recruiting the control group from a separate institution allowed for an ethically acceptable comparator while maintaining study integrity. After the intervention, the faculty implemented a follow-up program at the day center, providing supervised physical activity sessions three times per week. This ensured ongoing engagement in physical activity for control participants, supporting ethical principles and promoting long-term well-being, while highlighting the institutional commitment to older adults’ health (Figure 1).

A total of 34 physically inactive older adults were recruited and allocated into three groups: an active video game group (AVG; n = 12), a conventional exercise group (CEG; n = 11), and a control group that continued performing their usual daily activities (CON; n = 11). At the end of the intervention, only 24 participants completed more than 95% of the sessions and the pre- and post-intervention assessments of body composition, glucose, and lipid profile (AVG n = 8, CEG n = 7, and CON n = 9; Table 1).

An informational meeting was held with all individuals interested in participating in the physical activity program. Following the explanation of the study procedures, the Physical Activity Readiness Questionnaire (PAR-Q) was administered to determine whether participants were eligible to engage in physical exercise. Subsequently, the International Physical Activity Questionnaire (IPAQ) was applied to assess each participant’s physical activity (METs-min/week) and sitting time (min/day). Participants who met all inclusion criteria were provided with an informed consent form, which they were required to read and sign voluntarily.

### 2.1. Baseline Assessment

Participants were instructed to attend the Human Motor Skills Biosciences Laboratory following a minimum 10 h overnight fast and were asked to refrain from consuming food or beverages after 10:00 p.m. on the previous day. On the day of assessment, participants arrived between 8:00 and 9:00 a.m. Participants voluntarily read and signed the informed consent, the PAR-Q, and the IPAQ. Subsequently, height, weight, muscle mass, body fat, and phase angle (an indicator of cellular health reflecting cell membrane integrity and fluid distribution) were assessed using bioelectrical impedance analysis (InBody 770; Seoul, Republic of Korea). The evaluation began with the collection of a blood sample for glucose and lipid profile (total cholesterol, HDL, LDL, VLDL, and triglycerides).

### 2.2. Intervention

Participants in the AVGs and CEGs were asked to attend 24 exercise sessions, three times per week over a two-month period. Each session began with five minutes of warm-up, followed by 50 min of exercise, and concluded with five minutes of cool-down. For the AVG sessions, the video games Fitness Boxing 2 (Kyoto, Japan) and Ring Fit Adventure (Kyoto, Japan) for the Nintendo Switch (Kyoto, Japan) console were used. Fitness Boxing 2 is a full-body video game in which participants perform boxing movements (e.g., jab, cross, hook, uppercut, bob, and weave) using the free training mode, with progressive increases in movement complexity and session intensity [15]. Ring Fit Adventure is another full-body video game played in story mode, in which participants battle enemies through physical exercise, including exercises targeting the arms, trunk, and legs, as well as yoga-based movements [16].

All CEG sessions were supervised by professionals in physical activity and sport. The training was performed using only dumbbells, resistance bands, and medicine balls. The program consisted of eight exercises proposed by Cavalcante et al. [17]: (1) chest press, (2) leg press, (3) seated row, (4) knee extension, (5) preacher curl, (6) leg curl, (7) triceps pushdown, and (8) seated calf raise. Each exercise was performed in two sets at an intensity corresponding to 10–15 repetitions maximum (RM), with a two-minute recovery period between sets.

Exercise sessions in both the AVGs and CEGs were performed at a self-selected intensity. Exercise intensity was monitored by recording heart rate (pulse oximeter, Choicemmed, MD300C22; Beijing, China) and rating of perceived exertion [18] every five minutes during all AVG and CEG sessions. The maximum heart rate (HRmax) was estimated based on the formula: 208−0.7 × age [19]. No significant differences (*p* > 0.05) were observed between the reported intensity of AVG sessions (106.4 ± 16.2 bmp) and CEG sessions (106.0 ± 20.1 bpm).

### 2.3. Post-Intervention Assessment

Upon completion of the 24 exercise sessions (two-month intervention), participants from all three groups underwent reassessment of body weight, muscle mass, body fat, and phase angle. In addition, a blood sample was collected to determine glucose and lipid profile variables. Adherence to the intervention was defined a priori as attendance at a minimum of 95% of the scheduled sessions (i.e., at least 22 out of 24 sessions). Only participants who met this adherence criterion and completed both pre- and post-intervention assessments were included in the final analysis.

### 2.4. Statistical Analysis

Descriptive statistics mean and standard deviation (M ± SD) were calculated for all analyzed variables using the IBM-SPSS software, version 23. A two-way mixed ANOVA (groups [AVG, CEG, and CON] × time [pre-test, post-test]) was conducted for body weight, muscle mass, body fat, phase angle, glucose, total cholesterol, HDL, LDL, VLDL, and triglycerides.

Bonferroni post hoc tests were applied to follow up on statistically significant effects. Partial *eta* squared (η_p_^2^) was reported as a measure of effect size to describe the proportion of explained variance. Percentage changes (%Δ) between pre-test and post-test were calculated for each variable within each group. Statistical significance was set a priori at *p* < 0.05.

## 3. Results

### 3.1. Body Composition

**Figure 2:**
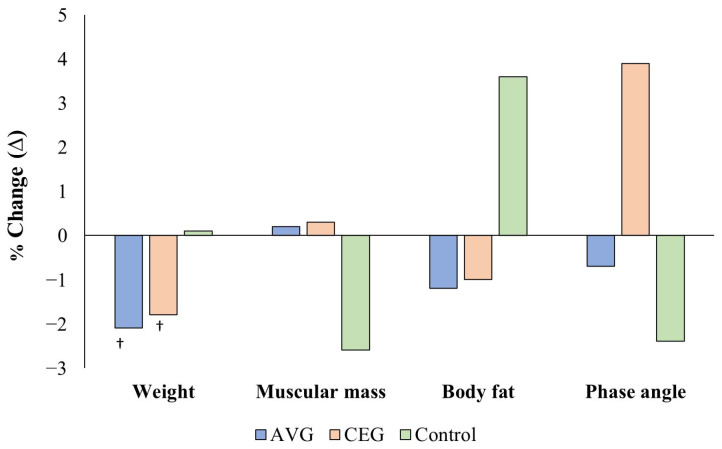
Percentage change in body weight, muscle mass, body fat, and phase angle in the active video game group (AVG), conventional exercise group (CEG), and control group (CON) after the intervention program. *Note*: ^†^ *p* < 0.05 indicates differences between pre-test and post-test.

No significant interaction between experimental groups and measurement times were found for body composition variables (*p* > 0.05). However, significant main effects were observed over time for body weight, as well as between-group differences for body weight, muscle mass, and phase angle (Table 2). The analysis revealed significant reductions in body weight from pre-test to post-test in the AVG (Δ = −1.3 kg, *p* = 0.013, η_p_^2^ = 0.26) and the CEG (Δ = −1.6 kg, *p* = 0.005, η_p_^2^ = 0.32), with no changes in the CON group (Figure 2). Regarding muscle mass, body fat, and phase angle, a significant increase in body fat was observed only in the CON group between measurements (Δ = 1.4 kg, *p* = 0.042, η_p_^2^ = 0.18).

Significant between-group differences were observed in body weight between AVG and CEG (*p* = 0.030) and between CEG and control (*p* = 0.017); in muscle mass between AVG and CEG (*p* = 0.001), CEG and control (*p* = 0.001), and AVG and control (*p* = 0.047); and in phase angle between CEG and control (*p* = 0.040). These findings indicate that the groups were not comparable at baseline in body composition variables.

### 3.2. Glucose and Lipid Profile

Significant interaction effects between time and group were found for total cholesterol (*p* = 0.001) and LDL (*p* = 0.009). Additionally, significant simple main effects of time were observed for blood glucose, total cholesterol, and LDL (Table 2). No significant differences (*p* > 0.05) were found for HDL, VLDL, or triglycerides across measurements in the AVG, CEG, or CON groups.

**Figure 3:**
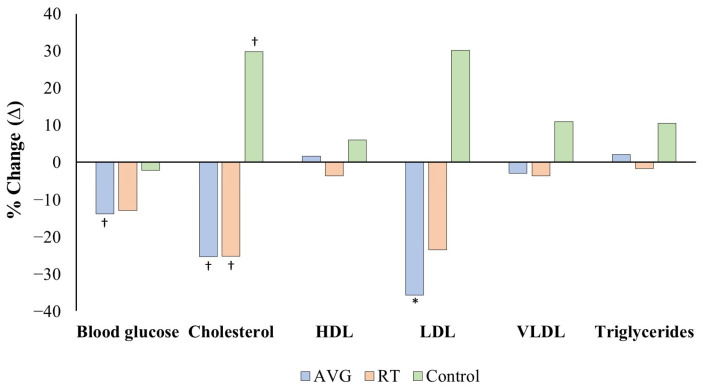
Percentage change (pre-test vs. post-test) in blood glucose, total cholesterol, HDL, LDL, VLDL, and triglycerides in the active video game group (AVG), conventional exercise group (CEG), and control group (CON) after the intervention program. *Note:* * *p* < 0.001; ^†^ *p* < 0.05.

The AVG demonstrated significant reductions after the two-month intervention in blood glucose (Δ = −16.9 mg/dL, *p* = 0.039, η_p_^2^ = 0.19), total cholesterol (Δ = −84.1 mg/dL, *p* = 0.002, η_p_^2^ = 0.37), and LDL (Δ = −83.9 mg/dL, *p* = 0.001, η_p_^2^ = 0.40). In contrast, the CEG showed a significant reduction only in total cholesterol (Δ = −56.7 mg/dL, *p* = 0.036, η_p_^2^ = 0.19). However, participants in the CON group exhibited an increase in total cholesterol (Δ = 47.9 mg/dL, *p* = 0.045, η_p_^2^ = 0.18) after two months of maintaining their usual daily activities (Figure 3).

Finally, significant baseline differences were found between the CEG and the AVG (*p* = 0.037) and CON groups (*p* = 0.016) in body weight, and between the CEG and the AVG (*p* = 0.001) and CON groups (*p* = 0.001) in muscle mass. Additionally, a significant baseline difference in total cholesterol was observed between the AVG and control groups (*p* = 0.015; Table 2). These findings indicate that the groups differed in body weight and total cholesterol at the beginning of the intervention program.

## 4. Discussion

The aim of this study was to determine whether two months of active video game training improves body weight, body composition (i.e., muscle mass and body fat), phase angle, and glucose and lipid profile compared with conventional resistance exercise and a control group of physically inactive older adults. Our findings were partially consistent with the initial hypothesis. Specifically, the reductions observed in blood glucose, total cholesterol, and LDL in the active video game group may suggest a potential beneficial effect of this modality on cardiometabolic health indicators. However, the hypothesis was not fully supported, as conventional resistance training showed changes limited to total cholesterol, and no significant modifications were observed in body composition variables across groups. These findings may indicate that the type of intervention could influence cardiometabolic responses, although other factors cannot be ruled out. Overall, active video game exercise was associated with reductions in blood glucose, total cholesterol, and LDL (and with the prevention of lipid deterioration observed in the control group), whereas the CEG was associated only with a reduction in total cholesterol.

One possible explanation lies in the nature of the stimulus generated by active video games. This type of intervention typically integrates dynamic movements, changes in pace, and intermittent aerobic components that increase cardiovascular demand and total energy expenditure [20,21]. These characteristics may promote activation of metabolic pathways associated with glucose uptake, such as contraction-mediated GLUT-4 translocation and activation of AMP-activated protein kinase (AMPK), thereby improving insulin sensitivity and contributing to reductions in blood glucose levels [22]. In contrast, conventional resistance training (particularly in short-term interventions) may produce adaptations more closely related to increases or maintenance of muscle mass rather than acute modifications in glucose metabolism.

Regarding the lipid profile, both programs achieved reductions in total cholesterol, which is consistent with evidence indicating that regular physical activity improves lipoprotein metabolism through increased lipoprotein lipase activity and greater peripheral utilization of fatty acids [23]. However, the additional reduction in LDL observed exclusively in the active video game group may be explained by a greater aerobic component and a longer accumulated time spent at moderate intensities, factors that have been associated with more pronounced improvements in reverse cholesterol transport and reductions in atherogenic lipoproteins [24].

Moreover, the playful and interactive nature of exercise through video games may have indirectly influenced the outcomes. This type of intervention has been shown to enhance motivation and reduce perceived exertion, potentially increasing adherence and actual exercise intensity during sessions [25,26]. In this study, however, exercise intensities between AVG and CEG were statistically similar (106.4 ± 16.2 bmp and 106.0 ± 20.1 bpm, respectively). In older populations, where adherence represents a determining factor in the effectiveness of exercise programs, this component may translate into a greater accumulated metabolic load and, consequently, broader cardiometabolic adaptations.

The simultaneous reduction in blood glucose, total cholesterol, and LDL observed in the active video game group has significant clinical relevance, as these markers represent independent cardiometabolic risk factors in older populations. Whereas conventional resistance training demonstrated a limited partial effect restricted to total cholesterol, active video games produced a more comprehensive modification of the metabolic profile. Furthermore, the increase in cholesterol observed in the control group reinforces the protective role of physical exercise against lipid deterioration associated with aging. The findings of the present study suggest that active video game-based exercise may represent an effective primary cardiovascular prevention strategy in older adults.

The comparison between active video game exercise and conventional resistance training should not be interpreted solely based on the average intensity achieved. Active video games are typically characterized by dynamic movements, motor variability, changes in pace, and intermittent components that induce greater cardiovascular and metabolic fluctuations. This variable nature of exertion may promote broader systemic activation, including more extensive cardiometabolic and neuromuscular responses. In contrast, conventional resistance exercise tends to generate a more localized and structured stimulus, with defined work and recovery periods, which may explain the differences in observed adaptations. Therefore, the quality and pattern of the stimulus—rather than isolated average intensity—may represent a key factor in interpreting the differential effects between both modalities in older populations [27,28].

The absence of changes in HDL, triglycerides, and VLDL suggests that the program did not provide a sufficient stimulus to induce more profound modifications in the lipid profile. In practical terms, increasing HDL and reducing triglycerides and VLDL generally require a higher volume of aerobic exercise, sustained moderate-to-vigorous intensity, and longer intervention durations (typically more than 12 weeks). Moreover, meaningful changes in these variables are often observed when exercise is combined with dietary modifications. Therefore, it is possible that a longer intervention period combined with dietary control would be sufficient to improve these lipid variables [24,29]. However, in the present study, we did not control dietary intake; thus, new protocols are warranted. These results indicate that a two-month active video game exercise program produces greater improvements in lipid profile variables compared with conventional resistance exercise. Furthermore, two months of physical inactivity increased total cholesterol levels in older adults.

In the study conducted by Mezghanni et al. [30], 31 young women with obesity were recruited and randomly assigned to one of three groups: moderate-intensity exercise (50% HRmax; n = 11), high-intensity exercise (75% HRmax; n = 10), and a control group (no exercise; n = 10), with the aim of evaluating changes in the lipid profile. Participants trained for 12 weeks (five times per week), with sessions lasting 20–25 min during the first three weeks, increasing to 40 min until week seven and 55 min thereafter. The researchers found significant reductions in body weight and body fat in both exercise groups; however, only the moderate-intensity group showed a decrease in LDL. The authors concluded that both exercise intensities were similarly effective in improving body composition, although moderate intensity was more effective in reducing LDL levels.

Rohmansyah et al. [31] conducted a similar study to compare the effects of high-intensity interval training (HIIT) versus moderate-intensity continuous training (MICT) on body composition and lipid profile in older women. The study included 24 women aged 50 to 60 years old, who were randomly assigned to either the HIIT group (n = 12) or the MICT group (n = 12). Both groups trained three times per week for 16 weeks; however, the HIIT group performed 38 min sessions at 90–95% HRmax, whereas the MICT group completed 47 min sessions at 70–75% HRmax. The researchers reported significant reductions in body weight (Δ = 0.7 kg) and body fat (Δ = 1.2%) in the MICT group, while the HIIT group showed significant reductions in body fat (Δ = 1.7%) and blood glucose (Δ = 0.6 mmol/L). The authors concluded that moderate- and high-intensity exercise similarly affect body composition and lipid profile.

In the present study, although a significant reduction in body weight was observed in both exercise groups (~2% of initial body weight), the magnitude of change was relatively small, which may explain the absence of detectable differences in muscle mass, body fat, and phase angle. In older adults, structural modifications in body composition generally require greater weight loss or interventions of longer duration and higher intensity. Furthermore, bioelectrical impedance analysis may not be sufficiently sensitive to detect subtle changes when total weight variation is moderate. Therefore, the observed reduction may reflect mild body adjustments that were not substantial enough to be expressed as significant changes in the evaluated body compartments. It is important to note that there is limited scientific literature evaluating phase angle and lipid profile responses following active video game-based exercise interventions, particularly when compared with other exercise modalities, despite the established clinical relevance of these variables in adults over 60 years [32,33,34,35]. These results indicate that two months of exercise, either through active video games or conventional exercise, reduced body weight. However, the bioelectrical impedance scale was not sufficiently sensitive to determine whether the weight loss was attributable to changes in muscle mass or body fat.

In addition to acknowledging the presence of baseline differences, it is important to consider how these initial disparities may have influenced the study outcomes. For instance, the higher baseline total cholesterol observed in the AVG may have allowed for a greater margin of improvement, potentially contributing to the larger reductions observed after the intervention. Similarly, differences in body weight and muscle mass between groups may have affected metabolic responses to exercise, as individuals with different initial physiological profiles can respond differently to the same training stimulus.

Participants were allocated into groups (AVG, CEG, and CON) based on recruitment source and availability to attend the intervention sessions, rather than through randomization. Specifically, participants in the control group were recruited from a senior day care center, while those in the exercise groups were recruited from a university-based physical activity program. This non-randomized allocation may have contributed to baseline differences between groups and is acknowledged as a limitation of the study.

Although the repeated-measures design partially accounts for within-subject changes over time, these baseline discrepancies should be considered when interpreting between-group comparisons. Therefore, the results should be interpreted with caution, and future studies using randomized allocation and larger sample sizes are warranted to minimize these differences and strengthen causal inference.

### Limitations

One of the main limitations of the present study was the presence of baseline differences between groups, specifically in body weight, muscle mass, and total cholesterol. These initial discrepancies may have influenced the magnitude and direction of the changes observed after the intervention, as participants did not start from equivalent conditions. Nevertheless, despite these between-group differences, the repeated-measures design allowed for the analysis of within-subject changes, partially mitigating the impact of baseline variability on the interpretation of intervention effects.

Another limitation was attrition, as 10 participants withdrew from the study for personal reasons unrelated to the study protocol. This loss may have reduced the sample effect size and, consequently, the statistical power of the analysis, in addition to potentially introducing bias if the characteristics of those who withdrew differed from those who completed the study.

Although some studies suggest that eight weeks of exercise (3 sessions/week, 60 min/session) is sufficient to induce changes in body weight and body fat [36], others indicate that a longer intervention is necessary to observe improvements in muscle mass and phase angle [10]. Therefore, extending the intervention to at least 12 weeks, while maintaining the same session frequency and duration shall be tried in future research.

The findings of the present study suggest that exergame interventions may represent a viable strategy in community settings, as they can be implemented with accessible resources and within relatively short time frames. Moreover, they could constitute an appealing alternative for older adults with low adherence to traditional exercise, since modalities such as active video games may enhance motivation and participation. These results may be relevant for the design of active aging programs, indicating that health benefits could potentially be achieved through adaptive and dynamic approaches. In this regard, the intervention might be integrated into senior day centers, universities, and municipal programs targeting older adults, thereby potentially expanding the available options to promote regular physical activity and well-being in this population.

When interpreting the present findings, it is also relevant to compare the effects of exergaming with other exercise modalities, such as high-intensity interval training (HIIT), which has been widely studied in older adults. HIIT is characterized by short bursts of high-intensity effort interspersed with recovery periods and has been shown to improve glucose regulation, lipid profile, and cardiovascular function.

Although the intensity profile of exergaming differs from traditional HIIT, both modalities share an intermittent and dynamic nature that may promote metabolic adaptations beyond those achieved through continuous or structured resistance exercise. However, unlike HIIT, exergaming may offer greater accessibility, enjoyment, and adherence, which are critical factors in older populations. These differences suggest that while HIIT may provide a potent physiological stimulus, exergaming represents a more feasible and sustainable alternative for long-term implementation in community settings.

## 5. Conclusions

Two months of active video game training may lead to greater improvements in lipid profile compared with conventional resistance exercise in physically inactive older adults. These findings suggest that active video games could be a safe and potentially effective exercise modality for supporting cardiometabolic health in aging populations, particularly in community-based settings where adherence to traditional exercise may be limited. From a practical perspective, exergaming appears to be a feasible and engaging strategy to encourage physical activity among older adults, with potential for implementation in senior centers, universities, and public health programs.

Future research should investigate the long-term effects of exergaming interventions using larger randomized samples, longer intervention periods (≥12 weeks), and controlled dietary conditions. Additionally, studies are needed to determine whether combining exergaming with nutritional strategies or other exercise modalities may produce more comprehensive improvements in body composition and lipid profile, including HDL, triglycerides, and VLDL. Further research should also explore the mechanisms underlying these adaptations and evaluate their impact on clinically meaningful outcomes such as cardiovascular events, functional capacity, and quality of life.

## Figures and Tables

**Table 1:** Participant characteristics by group.

	AVG	CEG	CON
Men/Women (n)	0/8	3/4	0/9
Age (yr.)	66.1 ± 5.0	65.9 ± 4.0	72.8 ± 2.0
Height (cm)	157.6 ± 5.2	164.0 ± 7.7	150.1 ± 7.0
Weight (kg)	64.8 ± 6.0	79.6 ± 14.7	63.2 ± 9.7
METs-min/week	1023.0 ± 670.8	903.3 ± 416.6	635.4 ± 437.2
Sitting time (min/day)	161.3 ± 67.9	194.3 ± 40.8	292.2 ± 116.8

Note: AVG: active video game group; CEG: conventional exercise group; CON: control group.

**Table 2:** Bioelectrical impedance and lipid profile values before and after a two-month program in the active video game (AVG), conventional exercise (CEG), and control (CON) groups.

	AVG		CEG		Control		ANOVA *p*-Value (Interaction, Measures, Groups)
	Pre-Test	Post-Test	Pre-Test	Post-Test	Pre-Test	Post-Test	
Weight (kg)	64.8 ± 6.0	63.4 ± 6.4	79.6 ± 14.7	78.0 ± 13.1	63.2 ± 9.7	63.1 ± 8.9	0.067, 0.002, 0.001
Muscular mass (kg)	21.3 ± 1.2	21.4 ± 1.3	27.3 ± 4.1	27.3 ± 3.9	18.2 ± 1.9	17.7 ± 2.0	0.062, 0.219, 0.001
Body fat (%)	38.5 ± 5.9	38.0 ± 5.5	36.6 ± 7.2	36.2 ± 7.0	43.9 ± 8.1	45.3 ± 7.5	0.087, 0.762, 0.062
Phase angle (°)	4.9 ± 0.6	4.8 ± 0.6	4.8 ± 0.7	5.0 ± 0.7	4.1 ± 0.7	4.0 ± 0.7	0.340, 0.974, 0.019
Blood glucose (mg/dL)	119.6 ± 10.6	102.8 ± 9.5	126.7 ± 16.0	110.0 ± 13.8	130.7 ± 31.4	122.9 ± 21.6	0.620, 0.005, 0.158
Cholesterol (mg/dL)	250.0 ± 74.8	165.9 ± 33.3	233.1 ± 50.0	166.1 ± 49.3	169.8 ± 22.6	217.7 ± 50.6	0.001, 0.035, 0.675
HDL (mg/dL)	63.4 ± 3.5	64.4 ± 5.0	63.6 ± 3.0	61.3 ± 7.8	61.7 ± 4.3	62.4 ± 3.2	0.099, 0.464, 0.816
LDL (mg/dL)	154.9 ± 70.2	71.0 ± 31.0	95.4 ± 65.9	67.9 ± 45.3	89.6 ± 18.5	112.4 ± 47.9	0.009, 0.034, 0.287
VLDL (mg/dL)	37.5 ± 12.2	35.6 ± 8.9	34.6 ± 11.6	31.6 ± 4.8	29.6 ± 3.9	32.6 ± 7.7	0.279, 0.703, 0.749
Triglycerides (mg/dL)	175.5 ± 48.8	177.3 ± 44.4	172.0 ± 57.1	162.6 ± 28.1	148.0 ± 19.6	163.6 ± 37.8	0.376, 0.718, 0.527

## Data Availability

The original data presented in the study are openly available in Open Science Framework at https://doi.org/10.17605/OSF.IO/9BEQZ (accessed on 19 March 2026).

## Abbreviations

The following abbreviations are used in this manuscript:

LDL	Low-Density Lipoprotein
HDL	High-Density Lipoprotein
VLDL	Very Low-Density Lipoprotein
AVG	Active Video Games Group
CEG	Conventional Exercise Group
CON	Control Group
PARQ	Physical Activity
IPAQ	International Physical Activity Questionnaire
AMPK	AMP-activated protein kinase
HRmax	Maximal Heart Rate
HIIT	High-Intensity Interval Training
MICT	Moderate-Intensity Continuous Training
